# The Power of the Soul over the Body

**Published:** 1845-10

**Authors:** 


I. THE POWER OF THE SOUL OVER THE BODY, CONSIDERED IN RELATION TO HEALTH AND MORALS. By *George Moore*, M.D. &c. 8vo. pp. 305. London, 1845. Longman & Co.

II. DES HALLUCINATIONS, OU HISTOIRE RAISONNÉE DES APPARITIONS, DES VISIONS, DES SONGES, DE L'EXTASE, DU MAGNETISME, ET DU SOMNAMBULISME. Par *A. Brierre de Boismont*, D.M. &c. 8vo. pp. 615. Paris, 1845. Bailliere.

III. RAPPORTS DU PHYSIQUE ET DU MORALE DE L'HOMME. Par *P. J. G. Cabanis*. Nouvelle Edition, par le Dr. *Cerise*. 12mo. pp. 567. Paris, 1843.

IV. THE ANATOMY OF SLEEP, &c. By *Edward Binns*, M.D. Second Edition, with Annotations and Additions by the *Earl of Stanhope*. 8vo. pp. 505. London, 1845. Churchill.

V. SEVEN LECTURES ON SOMNAMBULISM. Translated from the German of Dr. *Arnold Wienholt*, by *J. C. Colquhoun*, Esq. Advocate, author of Isis Revelata, &c. 12mo. pp. 219. Edinburgh, 1845. A. and C. Black.

THE several works, whose titles we have prefixed, embrace a variety of subjects that certainly have no very immediate relation the one to the other. They all, however, serve to establish or illustrate a great physiological and practical truth—the Influence of the Mind and feelings upon the state of the bodily Health; and it is with the view of drawing the attention of the reader to this important topic that we have coupled them together upon the present occasion. We gladly avail ourselves of this opportunity of offering a few remarks on a question that cannot but be interesting to every medical man, and to the undue appreciation of which may be traced, we believe, not a few of the fallacies, as well as difficulties, in the history and treatment of disease.

That the state of the mind and moral feelings has much to do with the induction, the persistence, and the final issue of many maladies, will not be disputed by any one, however ignorant he may be of the structure and functions of the body; personal experience too "feelingly persuades" him of its truth, and prevents him, alas! from remaining long in ignorance of the fact. There is a continual acting and re-acting of mind upon body, and of body upon mind. The one cannot suffer without the other immediately sympathising along with it. A fit of indigestion, or a twinge of the toothache levels the philosopher with the common herd of mankind; and, on the other hand, a feeling of shame will instantaneously crimson the cheeks; a sudden alarm will make the heart knock against the side and paralyse the limbs; intense grief will so wither up the frame as to blanch the hair in a single night; while, on the contrary, a noble effort of the soul has been known to bid defiance for a time to the severest agony, and put even a temporary check to the onward march of death. The physician, we need not say, has by far the most ample opportunities of studying this mutual and reciprocating relationship between the mental and corporeal parts of our frame. Not a day passes over without some example or another being brought under his notice : nay, there is scarcely a single case of disease that does not afford an illustration of its force, in a greater or lesser degree. The varying cares and troubles that attend every period of life from adolescence to old age ; the anxieties and turmoils of emulation, love, worldly business, domestic vicissitudes, thirst for praise, ambition, &c. and that host of minor sorrows and chagrins that follow so closely upon the steps of man—all tend to disarrange that exquisite harmony of combined functions that constitutes the state of health. And as the hardest rock may either be rent in pieces by the explosion of the ignited mine, or be gradually worn away by the ceaseless dropping of water upon its surface, so may the strongest frame either be shattered by the whirlwind of passion, or decay under the slow operation of incessant disquietude.

No author, in the present day, has written so ably or so effectively on this most interesting subject of enquiry as Dr. James Johnson. His works on Indigestion, Gout, &c. abound with numerous valuable remarks that well deserve attentive consideration ; but it is more particularly to his "Change of Air," and his "Economy of Health," that we would refer the reader for the most shrewd and practical observations that, as far as we know, are to be found anywhere. Their perusal will not fail to convince everyone of the high importance of attending to mental and moral agencies in the treatment of disease. Every year that we live, we not only see but *feel* the sound truth of this remark. The resources of medicine are not confined to the *Materia* Medica. Hippocrates must have thought so when he said—*Δει μεταγειν την σοφιαν εις την ιατρικην, και την ιατρικην εις την σοφιαν; ιατρος γαρ φιλοσοφος ισοθεος.* There is a *medicina mentis* as well as a *medicina corporis.* The accomplished physician will never neglect the use of moral therapeutics. His mission is of a higher character than that of being a mere prescriber from the formulas of the Pharmacopœia. If he cannot indeed "minister to a mind diseased," he may yet have it often in his power to soothe many of the secret sorrows and troubles of the heart. A kind expression, an unobstrusive enquiry, a word in season betokening interest and regard, may serve to draw out the real cause, and thus lead to the cure, of a pining sickness that had long resisted the ablest efforts of the mere practitioner. And never was it so necessary for medical men to act this benevolent and considerate part than in the present day, when there is so much mental disquietude among all classes of society, and among persons of all ages—owing doubtless to the increasing excitements of progressive civilisation, of misdirected education and discipline, of commercial vicissitudes, of political agitation, and of the numerous disturbing elements that spring from these sources. How strikingly is the truth of this displayed by the melancholy fact, that just in proportion as nations advance in outward prosperity and in intellectual refinement, so does the relative number of lunatics among their inhabitants seem to increase ! Is it not humiliating to think that there is a greater proportionate amount of Insanity in England, France, and America than in semi-barbarous Russia or in enslaved Turkey ?

But we must quit these generalities, and proceed to work out the theorem which we have proposed for consideration ; viz. the Influence of the Mind upon the health of the Body. In this enquiry, we shall first offer a few remarks "On the effects of mental attention on bodily organs"—a subject that has been *effleuré* rather than discussed by Dr. Holland, in his Medical Notes and Reflections.

It is a well-known fact that, if the attention be earnestly or for any length of time directed to any part of the body, a sensation of uneasiness and even of pain will almost inevitably be induced in that part. A toe may be made to ache, the nose to itch, the stomach to be affected with sickness, the bladder to discharge (or perhaps to refuse to discharge) its contents, and so forth, by merely thinking of, or fixing the attention upon, these organs. It is upon this principle that we can understand how it comes to pass that, in nervous and irritable constitutions, a pain once felt in a part is almost sure to return again and again, until perhaps it becomes fairly established, and—after continuing for days, or weeks, or even months, and resisting every remedial means that may be tried—will suddenly cease upon some strong impression being made upon the mind and feelings.

*Case.*—A young gentleman gave up his situation in a merchant's office in a pet. He soon became fidgetty and uncomfortable, not knowing how to occupy himself, and having no immediate prospect of employment. Before a week was over, he began to complain of a sharp pain in one of his knee-joints. It was leeched, fomented, and so forth, but seemingly to no purpose ; and, as he was of a scrofulous constitution, his medical attendant began to be somewhat uneasy about the complaint. It so happened, about this time, that this young gentleman's father had heard of a favourable opening for his son as a partner in a commercial house, and had made all the preliminary arrangements for the union. From the first intimation of this piece of good news, all pain in the knee ceased, and health was at once restored.

We shall give another illustration from our own experience.

*Case.*—A gentleman, aged about 30, and of highly sensitive feelings, had been for some years subject to severe attacks of Neuralgia in the left infra-orbital nerve. During this time he was a clerk in a writer's office in Edinburgh, but had been long anxious to leave it and come to London. At length he did so : and, although for several months his mind was kept anxious from the uncertainty of success, he never experienced any return of his suffering. He obtained employment abroad, and has now remained for several years quite free from pain in the part affected.

Cases of this description are most frequently observed in hysterical girls and women ; and we need scarcely say that the affection often assumes in them a most Proteian character. In the treatment of such maladies, the medical man who is bold and confident, and full of good promises to his patient, will succeed the best. Hence the frequent success of the empiric over the *regular*.

Mr. Quain once related, at the Westminster Medical Society, a curious case illustrative of the morbific effects of the attention being inordinately directed to a particular part of the body.

"A gentleman, who had constantly witnessed the sufferings of a friend afflicted with stricture of the œsophagus, had so great an impression made on his nervous system, that after some time he experienced a similar difficulty of swallowing, and ultimately died of the spasmodic impediment produced by merely thinking of another's pain."—*Moore,* p. 273.

In the following case, there was an exciting cause. A lady accidentally swallowed a plum-stone. No doubt it passed at the time into the stomach; but she persisted in believing that it stuck in her throat, in consequence of an uneasy feeling remaining in that part. The neck was accordingly leeched and poulticed, and a probang was passed several times down her throat!—as a matter of course, to her great inconvenience, and without any benefit to the guttural discomfort. It gradually became less and less and ultimately ceased; but it was from this date that her general health began to decay. Symptoms of heart-disease (which probably had been existing for some time) made their appearance; these were followed by dropsy, of which she died.

Dr. Moore attributes the curious phenomena in the case of Col. Townsend, who had the power of arresting the movements of his heart at will, to disease of that organ, under unnatural attention to its action. It may be worth while briefly to notice this remarkable instance of the influence of the mind over the body: it was originally related by Dr. George Cheyne.

"A Colonel Townsend, residing at Bath, sent for Drs. Baynard and Cheyne and a Mr. Skrine, to give them some account of an odd sensation which he had for some time felt, which was, that he could expire when he pleased, and, by an effort, come to life again. He insisted so much on their seeing the trial made that they were forced at last to comply. They all three felt his pulse which was distinct, and had the usual beat. He then composed himself on his back for some time. By the nicest scrutiny they were soon unable to discover the least sign of life, and at last were satisfied that he was actually dead; and were just about to leave him, with the idea that the experiment had been carried too far, when they observed a slight motion in the body, and gradually the pulsation of the heart returned, and he quite recovered. In the evening of the same day, however, he composed himself in the same manner and really died." P. 224.

There is not a part or organ of the body in which existing uneasiness or discomposure may not be aggravated or relieved, according as the attention is directed to, or diverted from, it. The function of Respiration is not a little affected in this way. Look at a person yawning, nay, only read about the act, and straightway you begin to imitate it. A fit of Asthma has been induced by merely seeing another person affected with it. Many obstinate and most distressing Coughs are kept up and aggravated by undue apprehension of their return.

*Case.*—A gentleman, 75 years of age, of intellectual habits and not mixing much in society, has for the last 25 or 30 years been subject to a violent tearing sort of a cough, which returns in frequent paroxysms of great severity; these paroxysms will sometimes last for nearly half an hour at a time. As a matter of course, the cough is always much worse if any cold is caught; but even in summer, it never fairly leaves him; more especially when he goes to bed, and when he rises in the morning. He has tried a host of remedies, and consulted at different times many medical men, all of whom have been agreed on this one point, that the lungs are not materially affected. His friends have always observed with pleasure that, whenever his attention in agreeably occupied, the cough has generally ceased to annoy him. Thus, although he has been more than usually harassed with it during the week, it disturbs him, on the whole, very little in church on Sundays. Pleasant company too has always the same good effect. Latterly, he has found a modicum of good whiskey toddy, taken at bed-time, have a decidedly quieting effect upon the cough.

*Case.*—A middle-aged lady, of a highly nervous and excitable temperament, has for the last twelve or fifteen years been distressed at times—more especially during the winter and spring months—with troublesome cough and most profuse expectoration, so as to confine her not only within doors, but even to her bed-room, for months at a time. We can give no exact particulars about the symptoms, having never seen her until the last few months; but we have heard that her friends have often believed that she was falling into a state of confirmed consumption. Her mind and feelings have long been kept in an anxious mood, in consequence of the ailing health and vexatious disappointments of her husband, a man of highly cultivated and impressionable mind. Latterly he has obtained a comfortable situation in a government office; and the consequence of this has been that her health has so much improved (without the aid of any medical treatment) that, during the whole of this most trying winter and spring (1844-5), she has been able to go out in almost all weathers.

These facts and remarks will perhaps be deemed sufficient, in proof of the position that we laid down as to the influence of "mental attention upon our bodily organs." We shall now go upon another tack, working our way, however, to the same point. Our present observations are intended to shew the *injurious effects of excessive study.*

Every one, accustomed to the labours of the closet, knows full well the tight uncomfortable sensation about the head that is apt to follow any unusually long or intense application of the mind. He feels as if a cord was passed firmly round the temples, or as if a weight was pressing upon the forehead right over the eyes. The sight becomes confused, and the hearing indistinct; and there is often such a bewilderment of his ideas that he scarcely knows what he is about. Spalding tells us that on one occasion, when his mind had been long kept on the stretch, he attempted to draw out a receipt. After writing two words, he found that he could proceed no further. For half an hour, he could neither think consecutively, nor speak, except in words which he did not intend. When he recovered himself, he found that, instead of writing on the receipt "fifty dollars, being half a year's rate, &c." he had written "fifty dollars, through the salvation of Bræ— ;" the last word being left unfinished, and without his having the least recollection of what he intended it to be. Sir Joshua Reynolds, after a long day's work in his studio, used not unfrequently to find, when he went out, that he saw trees in the lamp-posts, and moving shrubs in the men and women in the street. Dr. Watts tells us that, after prolonged study, he felt as if his head were too large to allow him to pass out of his room door; and a gentleman, after delivering a lecture at the College of Surgeons, said that it seemed to him as if his head actually filled the entire hall. Now in these, and in all such-like, cases, there must have been a very considerable disturbance in the equable circulation of the blood through the head, and consequently the incipient stage of a more serious lesion, had not the abnormal condition been timely removed by the suspension of the inducing cause. Hence it comes to pass that apoplectic and paralytic seizures are perhaps the most frequent causes of death in the case of studious and reflective men, more especially when they have passed the meridian of life.

Certain studies are, as a matter of course, more hurtful in their influence upon the brain than others. None are so injurious as abstract and metaphysical speculations, wherein the mind is intently occupied in attending to its own operations, or where one faculty is exercised, without relief or change, for an undue length of time. Dr. Moore has well observed on this subject, that " it would indeed appear that our Creator designed us to be employed rather on objects around us, and in association with the activities of other minds, than on the operations of our own; for we find that our efforts to concentrate attention on the process of our own thoughts speedily begets a most painful confusion; nor can we even summon our memory for the restoration of a forgotten idea and search with any diligence for its recovery, without such fatigue as either compels us soon to relinquish the pursuit, or else, if we obstinately persist, induces a nervous headache and imbecility, nearly approaching to aberration of intellect."

As bearing upon the same subject, we may here, *en passant*, allude to the excessively injurious effects of overstrained Education on the health of children and young people. One of the most common and hurtful practices of the present day is that of cramming the youthful mind with book knowledge, to the too general exclusion of that far more valuable instruction to be derived from Nature's own sweet page, and to the consequent neglect, at the same time, of proper attention to the state of the physical health. It may be laid down as an inflexible precept, that, while the body is growing, the mind should never be very much worked. This remark holds true more especially up to the 12th or 14th year of life; for, till that period, the size of the brain, and consequently the quantity of blood sent to it, are greater, in proportion to the rest of the frame, than at any subsequent period. If then, from any cause, this organ is unduly or overlong excited, can we wonder that the seeds of cerebral mischief are often laid, or, if not laid, are at least promoted to more active development, by the pernicious system of modern education? How general is the remark that Hydrocephalus, for example, is most frequent in those children, who are most clever at their books, and are therefore the pets of their parents.*

* M. Boismont remarks with perfect truth that misdirected education in youth is a frequent source of Hallucinations in after-life. The judgment and feelings are scarcely, if at all, disciplined; while the fancy and desires are indulged without moderation or controul. The nursery tales of ghosts and witches often haunt the dreams of the vacant mind, and thus serve to aggravate, if they do not induce, the delusions and aberrations of insanity.

The prematurely developed mind is too often the precursor—we might almost say, the sure harbinger—of early death. Do we not know that, if the tree begins to produce abundance of fruit, before it has attained its mature development, it soon exhausts itself and becomes, at best, unprofitable, if it does not entirely waste and wither away? And so it is with the complex machinery of the human frame. If the mind be overworked by excessive culture, while Nature is engaged in perfecting the body, the demand upon the energies of the system is too great; the one becomes exhausted, while the other is stinted of its fair proportions and dies.

There is good reason to believe that the state of the cerebral circulation is immediately and directly affected by every active emotion, and every strong effort of the mind. The quantity of the blood then sent to the brain is greater than it was before, and this increased flow continues, as long as the mental excitement lasts.

"Sir Astley Cooper had a patient, whose skull being imperfect allowed him to examine the movements of the brain. Sir Astleys says, 'I distinctly saw the pulsation of the brain was regular and slow, but at this time he was *agitated by some opposition to his wishes,* and directly the blood was sent with increased force to the brain, the pulsation became frequent and violent.' Dr. Pierquin witnessed the following case in the hospital of Montpellier, in 1821. Dr. Caldwell states that 'the subject of it was a female, who had lost a large portion of the skull and *dura mater* in a neglected attack of *lues venerea.* When she was in a dreamless sleep her brain was motionless; when her sleep was imperfect and she was agitated by dreams, her brain protruded from the cranium; in vivid dreams, *reported as such by herself,* the protrusion was considerable; and when perfectly awake, especially if engaged in active thought or sprightly conversation, it was greater still." P. 99.

Do not such facts as these, and the considerations which they suggest, most emphatically shew the first-rate importance of mental quietude in the treatment of cerebral disorders—more especially in those individuals in whom, from age or other causes, there may be reason to suspect a tendency to encephalic hæmorrhage? But it is not only the circulation in the brain that is affected; the *tone,* if we may so speak, of the entire cerebro-spinal axis is apt to suffer a simultaneous change. Any intense or long-continued application of the mind is invariably followed by an exhaustion of nervous energy, and a corresponding depression of spirits. Hence the irritability and restlessness that so generally accompany the poetic temperament. Byron has given, in his letters, a graphic account of the wretched hypochondriasis that he was apt to fall into, after an intellectual debauch. And who can doubt, after reading the life of Sir Walter Scott—whose natural character seems to have been far more equable than that of the *genus irritabile* generally—that the extraordinary efforts, which he voluntarily imposed upon himself after the occurrence of his misfortunes, shattered his mind, and aggravated, if they did not directly occasion, the cerebral disease of which eventually he died?

All imprudent thinkers, says Dr. Moore, are obnoxious to an accumulated irritability of brain.

"Even our great philosopher, Newton, sometimes gave vent to ill-temper or soothed his nerves by the bane of tobacco, instead of taking rest or appropriate change. And many of our best artists, whether in words or more solid materials, have been martyrs to head-ache and the fashion of excitement. Thus Wilkie was often obliged to shut himself up in a dark room, because light was too stimulant for his brain, and Paganini paid dearly for his consummate excellence as a musician. Speaking to a friend, he stated that he scarcely knew what sleep was; and his nerves were wrought to such almost preternatural acuteness, that harsh, even common sounds, often became torture to him. He was sometimes unable to bear a whisper in his room. His passion for music he described as an all-absorbing, a consuming one; in fact he looked as if no other life than that ethereal one of melody were circulating in his veins; but he added, with a glow of triumph kindling through deep sadness,—'*Mais c'est un don du ciel.*'" P. 249.

Such over-wrought excitement of mind not unfrequently terminates in positive Insanity. Swift himself predicted this woful calamity in his own case—to use his own forcible words, *that he would first die at top.* How striking the contrast between the latter end of the witty Dean and that of Dr. Wollaston! This truly Christian philosopher died of disease of the brain; and yet he preserved to the very close of his life the philosophic habit of observation which so eminently distinguished his character. "Sublime is the lesson," we use the words of Dr. Moore, "to see how he exercised the higher faculties of his intellect in reasoning on the causes and progress of his malady in the disorder of his sensations, memory, and the power of motion, as it advanced in its incursion upon one part after another of those portions of the brain which subserve the mind in relation to will and consciousness. He noted the phenomena of death, as it gradually took possession of his body, and experimented on his faculties to ascertain the amount of living power remaining."

Enough has now been said on this second argument of our subject. We shall therefore pass on to another, and seek our illustrations in that curious chapter of Physiology that treats of *the Effects of different Emotions upon Sensation.*

How much is the sharpness of our various senses affected by the mood in which we happen to be! When, from any cause, the mind is gloomy and depressed, our sensual perceptions are blunted or depraved; whereas, on the other hand, all gladdening emotions give strength and vigour to their exercise. Under the influence of joy, the eye sees more brightly; the ear hears more clearly; and the whole body is more quick and alive to external impressions than it was before. What says Shakespeare of the all-pervading influence of Love!

> "He, with the motion of all elements,
> Courses as swift as thought in every power;
> And gives to every power a double power,
> Above their functions and their offices.
> It adds a precious seeing to the eye:
> A lover's eyes will gaze an eagle blind;
> A lover's ear will hear the lowest sound,
> When the suspicious head of theft is stopped;
> Love's feeling is more soft and sensible,
> Than are the tender horns of cockled snails."

Those persons, in whom any of the senses is impaired or partially defective, can appreciate the influence of their feelings and state of mind on the acuteness of that sense's perceptions better than those in whom there is no defect. How often do we observe that this is the case with persons whose Sight or Hearing is somewhat at fault. Their infirmity varies not a little in degree at different times; they can see or hear much better on some days than on others, and in the company of those they like than in that which is unpleasant to them. All this is quite independent of the state of the atmosphere, or indeed of any external agency. The cause is in themselves. The grand secret with them is to keep the mind cheerful, and, at all events, tranquil; not over-anxious at the present moment, nor gloomily anticipating an increase of their infirmity, which they may never experience. One-half of human ills is in anticipation. As if not satisfied with present suffering, we perversely add to their amount by conjuring up those that are (perhaps are not) in futurity; and thus it is that, of our own influences, we make "coming events cast their shadows," not "before," as they ought to do, but "behind," darkening still more and more the already dim path of our journey through life. Horace was a wise man, if he practised his own precept:—

> "Tu, quamcunque Deus tibi fortunaverit horam,
> Gratâ sume manu; neu dulcia differ in annum."

The maxim is a golden one; would that we all followed it out better in practice.

Every deaf person will tell you that, whenever he is at all agitated, or more than usually anxious to hear, his Hearing is sure to be duller and more confused than ever. Does not this simple and most common fact show how much the activity of the sensual perception depends upon the state of the mind? A middle-aged gentleman, long resident in India, has been partially deaf for the last 15 or 20 years. As a matter of course he has consulted lots of aurists, and tried all sorts of remedies. It is now some eight or ten years ago since he first spoke to us on the subject. From a few observations that he made, we at once perceived that the deafness was in a great measure *nervous*, and therefore no doubt liable to frequent variations. He was advised to do nothing to his ears—the tympanum in one was broken—but attend to his general health and keep his mind easy. We met him the other day, upon his recent return from Calcutta, and were glad to find that he now hears better than he has done for several years past; although he has long ceased to use any local remedies for his deafness.

We cannot pass over this opportunity of denouncing the dishonest quackery that so generally prevails among most professed Aurists—what is the etymon of this word; *auris* or *aurum ?*—in the present day. Patients are fleeced of their guineas, visit after visit; their hopes all the while being kept up by very unfair promises of ultimate relief, until at length their purse, if not their patience, is worn out, and they are sent home not a whit the better, although their cases may flourish among the cures in the next yearly report of Mr. ——'s institution! So completely satisfied are we of the quackery that prevails in this department of the Healing art, that we do not hesitate to assert that not one case in a hundred of deafness, which has not yielded to the use of the most simple remedies—dropping a little warm oil into the ears at night, and syringing them with tepid water in the morning—is ultimately benefited by all the boasted refinements of Aural Surgery.

What we have said of the Influence of the mind and feelings on the senses of *Sight* and *Hearing* applies with still greater force to the exercise of our muscles under certain circumstances, when their action becomes irregular or impaired. Every one knows the perturbing effects of haste and passion on a *stammerer's* utterance; and the same remark applies to that peculiar affection of the hand that has been termed the *writer's cramp*, or stammering of the fingers. If the attention be directed to the suffering part, and the person be more than usually anxious to continue writing, he will inevitably find his annoyance to increase, until he is forced to abandon the exertion. (The reader will find the reports of several cases of this troublesome complaint in the Medico-Chirurgical Review, for July 1842).

Our *tactual sensations* and perceptions also are not a little influenced by the existing state of the mind. We shall allude to but one example, in the way of illustration of this. Who is there among our readers who has not found that he was much more readily *chilled* in a winter's day, when his spirits were low and languid, than when his heart beat high with joy and hopeful confidence? A mere effort of the will will do the same. Let him but whistle an air, or begin and spout a passage from a favourite author, and the want of the great coat is not felt so much as it was before. We read that Charles XII. at the siege of Frederickstadt, was in the habit of sleeping on the snow, wrapped only in his cloak, and that he bore extremes of cold, hunger and fatigue, under which numbers of his soldiers perished. They, poor fellows, had naught but their present sufferings to think of; while the mad monarch was all the while inflamed with the noble rage of ambition.

Dr. Darwin very happily illustrates the influence of the mind being amused in removing or preventing the sense of *fatigue*, by reminding us of what we often witness in a child. Although heartily tired, we have only to give the little fellow our walking-cane; he straightway plants it between his legs, and rides off upon it, as merry as a lark.

The same writer observes that he knew a gentleman, who told him that he was in the habit of relieving the feeling of weariness at any time by summoning up any angry idea in his mind. A joyful or a merry one, especially if it gave rise to a hearty laugh, would have answered quite as well, and would surely have been a much more pleasant remedy.

The *modus operandi* is obvious: a temporary energy is imparted by the mere effort of the will to the nervous system; and the immediate effect of this is to invigorate the exercise of the Respiratory and Circulatory systems —on the state of which the feeling of *fatigue* so much depends.

Take again the case of insensibility to *pain* under any strong mental excitement. A leg or an arm may be shot away during the mad fury of the fight, and the soldier will scarcely feel the pain of his wound until he has been carried off the field. The poor Hindoo will submit, at the bidding of his priest, to the most horrid inflictions of torture, and he seems to be almost insensible to his sufferings; and how often has the devoted martyr been known to bear unmoved the most revolting excess of cruelty from his persecutors! It is indeed astonishing what amount and duration of bodily suffering the human frame is capable of enduring, when the mind is worked up to a high pitch either of heroic resolution or of fanatic enthusiasm. The history of religion in all countries teems, alas! with too many examples of the truth of this assertion. Thank God, the spirit of the martyr often triumphs over the very sorest sufferings of its mortal tenement.

Of all the emotions of the human heart, none has a more animating and sustaining influence than Hope. It lightens the trials and afflictions of the present moment, and throws its cheerful radiance around the dark uncertainty of the future. Were it not for its blessed influence, the Body, as well as the Mind, would often sink under the sufferings and sorrows to which man is born, as the sparks fly upward. Many are the examples which we might draw from history, in illustration of its power in keeping up the energies of our bodily frame under all sorts of pain, privation, and disease. During the siege of Breda in 1625, when the garrison was on the point of surrendering from the ravages of Scurvy—principally induced by mental depression—a few phials of sham medicine were introduced, by order of the Prince of Orange, as an infallible specific. It was given in drops, and produced astonishing effects. Some of the soldiers, that had not moved their limbs for months before, were seen walking in the streets, sound, straight, and well.

Park tells us in his Travels that one day, in his journey through the burning desert, he laid himself down on the sand, exhausted with privations and fatigue, and ready, as he supposed, to die. He chanced at that moment to spy a tiny flower that had reared its head above the ground. What!—thought he—will that Providence which has watched over this humble plant, not care for me who has been taught to regard Him as a heavenly Father? The thought gave vigour to his drooping soul; and he immediately felt both his strength and his resolution fortified from that hour.

We read too that when Bruce, the hero of Scotland, was immured in a dungeon, at a time when all his prospects were most dark and unpromising, his mind was kept from desponding by watching the often repeated attempts of a spider to carry its thread from one point of the wall to another. Although often baffled, the creature persevered time after time, until at length it succeeded in effecting its wishes.

The account given by Xenophon of the sudden revival of his soldiers' spirits, when they first caught a glimpse of the sea, is so well known that it need not be particularised.

When we remember these and similar facts, can we wonder at the striking and immediate salutary effects which the sudden intelligence of good news will often produce on the result of many severe surgical, as well as medical, maladies? The patient straightway becomes fifty per cent. better. His pulse beats fuller and stronger, his breathing is more deep and regular, his eye glistens, and his appetite returns, and he looks as if suddenly relieved from the oppression of some morbific incubus. It is to this source that we must look for the cause of the marked contrast between the mortality among the wounded of a victorious, and that of a conquered, army? The most severe, and often too seemingly hopeless, cases recover in the one; while, in the other, Hospital Gangrene, Erysipelas, Typhus and Dysentery usually decimate the poor sufferers; and even the more favourable cases are both slow of recovery, and imperfect in their convalescence.

The sad history of our expedition to Walcheren affords a painful illustration of this fact. That to New Orleans might yield another; and, more recently, the disastrous retreat of our troops from Caubul offers a melancholy instance of the morbific effects of moral depression on the physical energies of our nature. Contrast the histories of these most painful events with those narratives of Byron's shipwreck, or of Captain Bligh's voyage in a boat across the Pacific—in both of which cases, despite the most fearful sufferings and privations, scarcely one man died from *sickness*—and you at once perceive the preservative and sustaining influence of mental and bodily activity, cheered by the "strength-inspiring aid" of Hope. But it needs not to turn to tales of "moving accidents by flood and field," to attest the power of this emotion on the body. Domestic life affords almost daily proofs.

Dr. Paris mentions a curious example of the salutary effect of confident expectation in the relief of disease. When the powers of the *nitrous oxyde* were discovered, Dr. Beddoes at once concluded that it must be a specific for *paralysis:* a patient was selected for the trial, and the management was entrusted to (Sir Humphry) Davy. Previous to the administration of the gas, the young chemist inserted a small pocket thermometer under the tongue of the patient, as he was accustomed to do upon such occasions, to ascertain the degree of animal temperature, with a view to future comparison. The paralytic man, wholly ignorant of the nature of the process to which he was to submit, but deeply impressed, from the representations of Dr. Beddoes, with the certainty of its success, no sooner felt the thermometer under his tongue, than he concluded the *talisman* was in full operation, and in a burst of enthusiasm declared that he already experienced the effect of its benign influence throughout his whole body: the opportunity was too tempting to be lost; Davy cast an intelligent glance at Mr. Coleridge, and desired his patient to renew his visit on the following day, when the same ceremony was performed, and repeated every succeeding day for a fortnight, the patient gradually improving during that period, when he was dismissed as cured, no other application having been used.

Dr. James Gregory used to relate in his lectures an instance of the power of the imagination in influencing the operation of a medicine, which may be here aptly introduced. One of his pupils, who laboured under fever, being unable to obtain any rest, was told that an opiate had been prescribed for him at bed-time; but the student misunderstood the doctor, and supposed that he was to take a purgative. Accordingly, when the physician saw his patient on the following morning, and inquired whether his opiate had procured for him any sleep, "Opiate!" exclaimed the patient, "I understood it was purgative, and very actively has it operated, and I am much relieved by it."

In strongly-attempered minds, a mere act of firm Resolution has been known to produce all the animating effects of joyful assurance. For example, it is related of Muley Molok that, in the last few hours of his life, upon hearing of the threatened defeat of his troops, he ordered his servants to carry him on his litter to the field: he calmly looked around, gave the necessary orders, and, then laying himself back, expired. Mr. Kingdon once related, at the Medical Society, a curious case that well displays the influence of a seemingly involuntary exertion of the mind upon the body, when labouring under disease. The patient, an old man, afflicted with shaking palsy, had been long unable to walk. The child of a friend was admitted to see him; and so greatly delighted was he, that he arose, walked across the room, took some paper, went to another part of the room, filled the paper with small shells, gave it to the child, and then sat down as paralytic as before!

We have known a result, somewhat akin to this, occur in a case of what may be called Hysterical Paralysis—in consequence, not of any act of the will, but of an unconscious effort during the excitement of delirium.

*Case.*—A middle-aged unmarried lady had been, for 15 or 16 years, confined to the bed and sofa, in consequence of the utter powerlessness of one of her lower extremities. A great number of medical men had been consulted, and the opinion of all was that no organic affection existed anywhere, and that the case was one of those anomalous and unmanageable affections which are apt to occur in females of a highly nervous and irritable character. During the delirium of an attack of fever, she arose from her bed—an effort which she had not made for upwards of fifteen years—and walked from one bed-post to another: this she repeated more than once, before her attendants replaced her in bed. On the circumstance being mentioned to us, we suggested the propriety of not checking, but of encouraging, her to walk about the room, in case she again rose. This plan was followed; and the result was that the patient ultimately recovered the use of her limbs so well as to be able to walk for one or two miles at a time. In this state she continued for two or three years; and then unfortunately gradually relapsed into her former state of semi-paralytic helplessness, in which she still remains.

We have heard of more than one similar case, in which a more complete recovery took place on a proposal of marriage being made to the long sofa-ridden invalid; and probably all have read of the miraculous (!) cure, some years ago, of a young lady at Camberwell, in answer to the prayers of Prince Hohenloe. It is in reference to such cases as these that Sir Benjamin Brodie has acutely remarked that it would seem as if it was rather the will than the muscles that were paralysed. We need not add that the lesion is one of function and not of structure. The throat and organs of speech are sometimes affected in this manner. Many cases of Hoarseness, and even of complete Aphonia, appear to be of a purely nervous character, and have therefore been known to get well on a sudden by a strong impression being made on the mind. "A young lady," (we quote from Dr. Darwin) "who had for many months been affected with an almost complete aphonia, and had in vain tried a variety of advice, recovered her voice in an instant, on some alarm as she was dancing at an assembly. Was this owing to a greater exertion of volition than usual?—like the dumb young man, the son of Crœsus, who is related to have cried out, when he saw his father's life endangered by the sword of his enemy, and to have continued to speak ever afterwards."

We may here remark that, even in cases of confirmed Paraplegia, it is well known that (generally at least) there is no organic change either of the muscles or of the nerves of the palsied part: all, that is really deficient or wanting *there*, is that the will has ceased to have the power over the affected limb or limbs, in consequence, it may be, of a lesion or disturbance in some part of the cerebro-spinal axis. Not only may the affected muscles be stimulated to action by the influence of Electricity and Galvanism; but, even in some ordinary natural acts, they are called into action, and contract as in health. Thus, in yawning, sneezing, and such like acts, the paralytic member will join in the general consentaneous movements of the body, although it may be wholly disobedient to the mandates of the will. Such movements must indeed be considered as involuntary; and yet a strong effort of the mind has been known to produce the same results. It is Sir Charles Bell, we believe, that remarks that he had seen a case where a woman, who had lost the use of both arms, was able to hold up her child as long as she kept her eyes fixed upon it; but, as soon as she ceased to look at it, or have her attention in any way diverted, she would immediately let it fall; she did not feel the pressure of the weight upon her muscles. We were recently consulted by a woman, who was subject to violent headaches, and attacks of sudden insensibility and loss of consciousness. Among other symptoms, she mentioned that, if she began to laugh, or be in any way excited, when she had anything in her hands, she was forced to let it drop, her arms losing all power of support; although, at other times, she could carry the dishes up stairs with perfect ease and safety. But we must stop this digression, and return *à nos moutons:* the emotion, that we shall next allude to, is Fear.

If Hope has an invigorating and animating effect upon the system, this, it is well known, is as powerfully depressing upon all its energies. The whole muscular system, involuntary as well as voluntary, is relaxed and unstrung; the skin is chilly damp, and the body is unable to generate its accustomed amount of heat; the circulation is hurried and irregular, and the blood is unequally distributed; the breathing is short and rapid, or takes place in intermitted deep-drawn efforts; and the nervous system—at least that part of it which supplies the organs of sensation—is in a state (but only for a time) of exquisite and overwrought tension—to be soon followed by one of relaxation and exhaustion. Such are the immediate effects of strong Fear, or Terror, upon the body; those produced by the emotion when less intense, but longer continued, are of the same general character—essentially depressing and enervating. We cannot therefore be surprised at its morbific influence. A person has actually been known to have been *talked* into a state of positive and eventually serious, and even fatal, illness. Lord Stanhope gives the following instance:—

"An English physician, while walking with one of his friends in the country, and conversing with him on the effects of imagination, observed a labourer who was advancing towards them, and said, 'That man enjoys excellent health and strength, and yet I could convince him that he is very ill.' This was doubted by his companion, and it was determined to make the experiment. The labourer was accosted by the physician, whom he knew to be such, and questioned about his health, but when he gave the most satisfactory answers to the inquiries, they seemed to excite much surprise, and he was told gravely, and with a sort of affectionate interest, that he was certainly in a state of disease which required great care. The labourer immediately returned home and went to bed, and replied to his wife, who endeavoured to persuade him that he was in his usual health, 'The doctor says that I am not, and he knows best.' As soon as the physician learned the unpleasant consequences which had ensued, he went to the house of the labourer, and apologised for them, assuring him that he was in perfect health, and that what had been said to him was merely to show the effects of imagination. Nothing that he could urge was of any avail, the labourer persisted that he was very ill, refused to leave his bed, and died in a few days."—*Binns*, p. 484.

Among other effects of this passion, we may remark that it renders the system unusually susceptible of the morbific influence of contagious fevers. The vulgar are surprised at the confidence with which medical men approach patients labouring under the most malignant diseases. Not a few, indeed, of our brethren have fallen victims to their devotion; but many more would unquestionably suffer, if the feeling of dread or apprehension entered their minds. Whenever the energies of the body are reduced—be the cause what it may—below the healthy standard, it immediately becomes less able to resist the depressing effects of a malarious poison. The vigour of the system therefore must be sustained, not stimulated, when a person is exposed to such an influence; and certainly there is no cordial more preservative than the animating influence of Confidence and Hope.

Fear very generally produces a relaxation of the sphincters of the mucous passages; hence Enuresis, Diarrhœa, and Abortion are often caused by it. We need scarcely add that Palpitation of the Heart is a very common consequence of this emotion. But fear has not always a morbific effect; it may exert the very opposite, a curative, one. Various Mimotic or imitative diseases have been checked by appealing to its influence. Boerhaave tells us that he once had a number of patients seized with epileptic fits in an hospital, from sympathy with a person who fell down in convulsions before them. He was puzzled at first how he should act; but, reflecting that the attacks were produced by a mental impression, he resolved to try the effect of making a still stronger impression to check them. Accordingly he ordered, in the hearing of the patients, hot irons to be prepared and applied to the first person who was seized. The consequence was, that no fit occurred afterwards.

An officer in the Indian army was confined to bed by asthma; he could only breathe in the erect posture. A party of Mahrattas broke into the camp; the invalid sprung out, mounted his horse, and used his sword with great execution. A lady, affected with hysterical semi-paralysis, had been confined to her bed and sofa for years, when a fire broke out in the house where she resided. The hitherto helpless creature rose up, rushed out of the room, and reached the street ere she was aware of what she had done. Hildanus relates that a man, disguised as a ghost, took another, labouring under severe gout, from his bed and carried him down stairs, and there left him. The terrified patient quickly found his way up again, and never afterwards had the gout.

The following extract is from Dr. Watson's Lectures on the Principles and Practice of Medicine.

"Strange stories are recorded—strange, but I believe true—of instantaneous cures of the Gout by strong mental emotion, by sudden terror, by violent wrath. Dr. Rush relates an instance of this. An old man, who for several years had suffered an annual attack of gout, was lying in one of these paroxysms, when his son, by some accident, drove the shaft of a waggon through the window of his room, with vast noise, and a great smashing and destruction of the glass. The old man leaped out of bed, forgetting his crutches; and his wife, on entering the apartment, was surprised to see him walking up and down, and exclaiming angrily against the author of the mischief. The late Professor Gregory, of Edinburgh, was in the habit of mentioning another example to the same effect, authenticated to him by a naval surgeon. It occurred in the person of an officer who was freed from an attack of Gout, when at sea, by an alarm of fire. Whether this influence of certain states of the mind be rightly alleged or not, it is clear that we can never hope to make any practical use of such a remedy. Indeed, a fit of the gout has been sometimes *brought on* by a mental shock."

The influence of Fear, or rather Disgust, in checking bleeding from the nose and other parts, is well shewn by the common success of a vulgar remedy, the putting of a living toad around the neck. Certain *nævi materni*, and such-like spots, are said to have been removed by the application of a dead man's hand to the part. Whether this be true, we shall not affirm; but there can be no doubt that the disgusting remedies, which at different times have been recommended to expedite Delivery, could not fail to have a decided effect by producing nausea and shuddering. A draught of the husband's urine has long been a favourite remedy in some parts of Germany; and Hartman says that horse-dung steeped in wine is very efficacious in promoting the expulsion of the placenta!

None of the Emotions has a more decided or more pernicious influence on the body than Anger. The violent tension of the muscles in different parts impeding the free course of the arterial circulation, and the disordered breathing disturbing the easy transit of the blood through the lungs, the current is necessarily thrown back upon the heart; and this organ, in consequence, labours with inordinate and irregular power to propel it with redoubled force. Hence it is that, under its violent influence, a blood-vessel not unfrequently gives way at some part, or even "the golden bowl (itself) is broken at the fountain."

A gentleman, while engaged entertaining a number of friends in his grounds, stamped his foot in anger at one of his servants. The immediate effect was to bring on an attack of Hæmoptysis, which laid the foundation of his fatal illness.

John Hunter attributed the commencement of his heart-disease to a fit of passion. And, indeed, when such disease is once fairly established, a patient cannot be too much on his guard against the excitement of all strong emotions. Many a person has expired in the very act of execration.

"Broussais and other eminent physiologists are of opinion that rage is capable of generating a most virulent and subtle poison, especially in the saliva. They refer to numerous instances, in which wounds from enraged animals have been followed by effects only to be accounted for by supposing a virus communicated. This opinion coincides with vulgar belief, and if true, as facts seem to affirm, the power of the mind in altering the chemistry of life in a direct manner is thus most clearly demonstrated. But indeed the same fact is equally evinced by the common influence of emotion over secretion. The classical reader will remember Ovid's fine description of Envy.

> " Pallor in ore sedet; macies in corpore toto;
> Nusquam recta acies; livent rubigine dentes:
> Pectora felle virent; lingua est suffusa veneno." *Moore*, p. 263.

Anxiety and Grief are allied in their effects to Fear, and, like it, have a signally depressing influence upon all the functions of the system. The muscles lose their wonted tone, and the nervous system its accustomed energy; the blood moves languidly and does not generate the due amount of animal heat; the appetite fails, and the bowels act sluggishly; the urinary secretion is usually abundant and pale, and the generative organs are languid and relaxed.

As these emotions therefore have a truly withering influence, seeming, as it were, to dry up the very sap, and enervate all the energies of life, can we wonder at such maladies as Dyspepsia, Jaundice, Hæmorrhoids, and even Diabetes and Paralysis, being not unfrequently traceable to such exhausting states of mind and feeling? The diseases now mentioned are all of a more or less distinctly chronic nature; a circumstance indicating that the operation of the obnoxious influence has been slow and long-continued. But it may be otherwise; its effects may be immediate.

The bustle of over-Anxiety, as indeed of every restless feeling, has been known to cause sudden death in cases of cerebral or cardiac lesion. A metropolitan hospital surgeon, a year or two ago, fell down in Garraway's auction room, where he had gone to *bid* for a property to be sold. A clergyman, anxious to return home to see his wife lying dangerously ill, expired immediately after he had pronounced the blessing.

Numerous forms of nervous *malaise* may generally be traceable to an over-anxious and irritable state of mind. Medical students know the truth of this in their own experience, when preparing for their examinations: heart-disorder is very common among them.* Dr. Binns relates an amusing instance of a fellow-collegian working himself into the belief that he was labouring under an aneurism of the left subclavian artery, in consequence of hearing a snap in that shoulder, while using the dumb-bells, and of a slight fulness of the left pectoral muscle supervening thereupon.

An attack of Jaundice has been known to be brought on from over-anxiety; but this disease is perhaps more frequently the effect of a fit of anger or of sudden chagrin. Mr. North witnessed a case of this kind, in which an unmarried female, on its being accidentally discovered that she had had children, became jaundiced almost immediately afterwards. The same effect, we may remark, has been produced by intense bodily suffering, by the pain of a severe surgical operation, or perhaps by the dread which

---

* Dr. James Johnson remarks, that " Diseases of the Heart were so little attended to before the French Revolution, as to be scarcely noticed by medical writers. The portentous scenes of that eventful period called forth such a multitude of this fatal disease that a volume was soon written on the subject by Corvisart—and the mental excitation, that has ever since continued, has kept up the tendency to affections of the Heart, which are now among the most prominent and dreadful of human afflictions."—*Economy of Health*, p. 96, 4th Edition.

preceded it. The functions of the Chylopoietic viscera are among the first to suffer on all occasions, when there has been severe mental or bodily disquietude. The disorder may not shew itself at the moment, or even for several weeks afterwards; but it almost inevitably supervenes; and the patient will then tell you, on enquiry, that he has not felt entirely well for some time before. This is a common occurrence among those who have been watching over the sick-bed of a dear friend; the fatigue of body and anxiety of mind seldom fail of inducing some visceral derangement or another. No remedy, we need scarcely say, is so good as change of air.

Of the digestive viscera, none is so immediately affected as the Stomach itself. The condition of this organ—the "conscience of the body," as it has been happily termed—is modified by almost every impression that is made upon the mind and feelings. The sight of any loathsome object will often excite nausea and sickness; and, if food has recently been taken it will remain undigested; for it would seem that the secretion of the gastric juice has been suspended or vitiated. Again, every one knows that Fear or Grief will at once take away all appetite, however keen it may have been a moment before. Over-anxiety will do the the same; and is not this, we may here enquire, the prolific cause of Dyspepsia among our mercantile and professional classes? For six or eight hours at a time, perhaps not a morsel of food is taken, and no *besoin de manger* is felt. Then, when the labours of the day are over, they hurry home to a late dinner, eat perhaps voraciously, drink more wine than does them good, and fall asleep on the sofa, to leave the stomach to do its work. The hurtful effects of such a mode of life may be slow of operation, and perhaps will not be decidedly experienced until the person begins "to go down the hill," and to feel that his strength was not what it used to be, or his powers of endurance so great as they once were.

Many of the ailments and diseases of the Climacteric period of man's life—the sixth decenniad, or the transition period (at least in most persons) from mature manhood to old age—are unquestionably referable to the state of anxious feeling and mental disquietude that so often occupy the mind at that time. Both mind and body are now losing, if they have not already lost, the energy and elastic spring of bye-gone years; we feel less aptitude for exertion, especially in any new line of occupation; and hence, if any mishap occurs in our affairs, we have not the resolution to bear up against it, or to apply ourselves bravely to retrieve the lost ground. Our more youthful fellow-labourers start in before us, and, by their superior activity and less hesitating cautiousness, win and keep the position which we formerly occupied. Besides, how often do domestic cares and sorrows thicken and abound at this period of a man's career! His children are grown up, and must now be disposed of. If they have been expensively educated, they look to their father, as a matter of course, for fortunes to start them in life; and, even in the more humble stations of society, some assistance will be expected by the daughters upon their wedding, and by the sons on entering upon business. Is it not too at this very period of a family's history that the waywardness, if not the positive viciousness and profligacy, of some one member is apt to be most severely felt by the father, who perhaps may have built his hopes of future happiness and prosperity upon the welfare of this very prodigal? We cannot therefore wonder much at the marked change and incipient breaking-up of the bodily health that is so often observed to take place in the course of the sixth decenniad of life among men, still engaged in business and exposed to the vicissitudes of commercial or professional employment. There is a care-worn expression of countenance; he loses flesh; his spirits flag, except perhaps in company, and then they often brighten up to almost youthful energy upon bye-gone topics; his appetite is not so keen and vigorous; the bowels act less regularly of themselves; there is very often a tendency to hæmorrhoids, and in short to venous fulness generally, more especially of the cerebral and portal systems: hence the torpor and tendency to drowsiness in some cases, and to hypochondriasis and querulous malaise in others. The mind has lost the elastic spring of more youthful years, and has not acquired the apathy of old age. Thus it is that—from some worry or vexation, or, it may be, from some slight bodily indisposition, which would have passed away in former years without any inconvenience—a certain degree of feverishness of the system is kept up; the wasting process exceeds the nutrient and reparative ones, while the need or *besoin* for the latter is still felt; the balance between the venous and the arterial circulation is materially modified; and the brain and nervous system are not capable of the same amount of expenditure of functional energy. Can we wonder then that Apoplexy and Paralysis are so frequent at this age? Not a few between 50 and 60 years of age are carried off by these diseases.

If the system holds out until the next decenniad of life begins, when old age has quieted down the irritability and blunted the overstrained sensibilities of our nature, the bodily health becomes often very strikingly improved, and the remaining portion of life is passed much more smoothly and equably than the ten or twelve years that had preceded the change. The feelings are now much less excitable; the chagrin of disappointed hope or ambition does not sink so deeply; and a certain degree of apathy creeps on, rendering the individual more submissive to the present, and less anxious about the future. In conclusion, we may remark, that the "climacteric disease" is much less frequent in women than in men; indeed it is scarcely known among the former.* The cause is obvious; the female mind is less exposed to the sources of anxiety and disappointment; and, moreover, it is unquestionably stronger and more patient under affliction than the spirit of the male.

Before quitting this part of our subject, it may be worthy of notice, that the sudden suspension of long-continued anxiety has been known to induce positive Insanity.

*Case.*—A middle-aged gentleman, who had, for upwards of fifteen years, been living on borrowed money in expectation of his father's fortune, at length came rather suddenly into possession of it. His affairs were considerably embarrassed at the time; and, for a year or two before, he had become more than usually anxious about the result. It would seem as if his nervous system had, like a musical cord, been wrought up to the

---

* The *life* of a woman at this period—the sixth decenniad—is, in the estimate of life-insurance companies, considerably *better* than that of a man.

highest degree of tension, so that any impression on it would cause it to snap. From the moment that he heard of his father's death, he manifested signs of mental derangement; his ideas running altogether on the subject of his fortune. He fancied that his wealth was boundless, and he was continually offering to write cheques for any sum desired to every person that he happened to meet.

But intense Grief is a much more frequent cause of mental alienation.

*Case.*—An elderly lady, who had concentrated all her hopes and affections upon her grandson,—her only son having died many years before—was doomed to be deprived of this darling of her heart by a sudden epileptic attack: he was found dead in his bed one morning. Within two or three months of his majority, every thing had promised good fortune to him, and happiness to his aged relative in witnessing his prosperity. Her grief upon his loss was most overpowering. She sobbed and wept like a child, who had just lost its mother whom it had learned to love and adore. Day after day passed over, and scarcely any abatement of her grief could be perceived. Gradually indeed it became more silent; but then it was too visible that her mind had become shaken in the conflict, through which it had passed. Her only conversation was about her dear boy; she wrote his name on every scrap of paper she could find; and, as she repeated it, she wept afresh. Then she began to believe he was not dead, and that some one kept him away from her. In this sad state, she remained until the mind, becoming more and more weakened, has at length lapsed into imbecility and utter childishness. Such are the effects of loving any earthly object "not wisely, but too well." It would be easy to multiply instances of the withering and wasting influence of intense Grief on the sanity of the mind as well as on the health of the body. Who has not read the touching story of the "Broken Heart" in the Sketch Book?

As a contrast to the slow and more gradual effects of this emotion, we shall now give an instance where they were instantaneous, but less permanent and abiding.

*Case.*—A young lady had the misfortune to lose her husband at sea, during their voyage from New South Wales to this country. She was pregnant with her first child at the time; and, as there was no other female on board, her condition for nearly three months was most truly distressing. On the vessel arriving in the river, the brother of her late husband went immediately down, and fetched her to apartments which he had provided: we need scarcely say that her grief at meeting was intense. No sooner had she stepped into the room, than she uttered a scream, and would have fallen on the ground, had she not been caught in the arms of the attendants. She was utterly insensible, and in this state continued when a medical gentlemen in the neighbourhood was called in. He ordered the hair to be cut off, and leeches applied to the temples: this was between 2 and 3 P. M. I did not see her until about six o'clock: she was still quite unconscious, and could not be roused to answer any question. As the face was pale, and the pulse feeble, I had her at once removed to bed, warmth applied to the feet, and a mustard poultice to the region of the heart: a tea-spoonful of brandy was attempted to be given, but she could not swallow. There was no symptom of revival, when I left about 8 o'clock. On returning about 11 o'clock, I found her in the same unconscious state, and—judging at least from the state of the pulse—decidedly much more exhausted, and indeed, to all appearance, rapidly sinking. I sat, with two friends of the patient, beside her bed for three hours, expecting her speedy dissolution; the pulse was scarcely to be felt; the breathing was very indistinct; and a death-like pallor was diffused over the surface. About 2 P. M. a change however began to manifest itself. We succeeded in getting a few spoonfuls of brandy and water down; and, as this was swallowed, the pulse rose and the skin began to regain its warmth. Between five and six o'clock, she became restless, and every now and then moaned, as if in pain. An examination was made, and the *os uteri* was found to be as large as a crown-piece. From this time, the labour-pains returned with tolerable regularity; so that by 4 P. M. the head was fairly in the hollow of the pelvis. Cephalotomy was performed, and the child extracted: it had probably been dead for 24 or 48 hours. During all this time, the poor sufferer was quite unconscious, save to the bodily pain of childbirth. Fortunately she slept, after the delivery, at intervals, and repeatedly took food. Her consciousness returned gradually; but the mind for several days was dwelling almost continually upon her lost husband, who she would not—every now and then—believe was dead. Her recovery was slow, but ultimately complete. Here then was a case of a condition approaching to that of a *trance*, induced by the outburst of long-smothered grief. Any other violent emotion is apt to do the same in females of a highly nervous and excitable temperament. We might adduce many examples in proof of this; but the only case, that we shall mention at present, is one of Cataleptic trance, related by the late Dr. Gooch, and which occurred in a woman a few days after her delivery of a dead child.

* * * * "She was lying in bed, motionless, and apparently senseless. Her eyes were open; but there was no movement of the chest, no movement of the nostrils, no appearance of respiration could be seen; the only signs of life were her warmth and pulse; the latter was, as we had hitherto observed it, weak, and about 120; her fæces and urine were voided in bed.

"The trunk of the body was now lifted, so as to form rather an obtuse angle with the limbs (a most uncomfortable posture), and there left with nothing to support it. Thus she continued sitting, while we were asking questions and conversing, so that many minutes must have passed.

"One arm was now raised then the other, and where they were left, there they remained; it was now a curious sight to see her, sitting up in bed, her eyes open, staring lifelessly, her arms outstretched, yet without any visible signs of animation; she was very thin and pallid, and looked like a corpse that had been propped up, and had stiffened in this attitude. We now took her out of bed, placed her upright, and endeavoured to rouse her by calling loudly in her ears, but in vain; she stood up, but as inanimate as a statue; the slightest push put her off her balance; no exertion was made to regain it; she would have fallen, if I had not caught her.

"She went into this state three several times. The first time, it lasted fourteen hours; the second time, twelve hours; and the third time, nine hours; with waking intervals of two days after the first fit, and one day after the second."

That *religious Enthusiasm* is one of the most powerful of the predisposing or even of the exciting causes of Cataleptic and Ecstatic seizures, is expressly stated by every enlightened medical writer on the history of nervous diseases. What says Dr. Copland ?

"These affections are most commonly excited by * * * * particularly religious enthusiasm ; great mental application, and the passion of love ; frights, terror, or uncommon dread ; the irritation of worms in the primæ viæ : suppression of the menses, of eruptions and accustomed discharges ; injuries of the head ; concealed mental emotions, and ungratified passions ; and disturbance of the uterine function. * * * * * I believe that many cases in females are chiefly exalted or more severe states of Hysterical affection, and more or less connected with disorder of the nerves and circulation in the uterus and ovaria."

Keeping these observations in view, we now request the reader's attention for a few minutes to certain cases of what Dr. Binns calls "Seraphic ecstacy :" their histories are related in a pamphlet published two or three years ago by that pious nobleman, Lord Shrewsbury.

The name of the first of the Earl's wonderful girls is Maria Morl, surnamed the "Estatica." She was born in 1812. In early youth, there was no peculiarity about her; she was like any other girl, only more than usually religious; for she was much in the habit of visiting a church of the Franciscans, near her father's house, for prayer. As she grew up, she became sickly and delicate ; but we are not told whether she had ever any epileptic or cataleptic seizure. It was not until she had reached her twentieth year, that she exhibited any signs of Ecstasy; and then, it would seem, the attack returned every time that she received the sacrament. As she usually remained in the ecstatic state for seven or eight hours at a time, her confessor very considerately thought that she would remain longer, if the "host" was given at a very early hour. Accordingly he administered it to her one morning at 3 o'clock, A.M. ; she immediately became entranced, and continued in the kneeling attitude of prayer for 36 hours ! The priest, then returning, found her in the same position that he had left her in ; and he, good man, felt convinced that she might continue so, for the rest of her life, "unless," we use Lord S.'s words, "he should bring it (the ecstatic condition) within limits, by recalling her to herself. He therefore undertook to regulate this state by virtue of that holy obedience, which she had vowed upon entering the third order of St. Francis !" Crowds of people came from the country to see this highly-favoured lady ; and indeed such throngs assembled that the authorities of the place thought it necessary positively to prohibit their meeting.

In the course of the next year, a sanguineous spot, like the scar of a recent wound, was observed on the palm of each of her hands. The priest at once predicted that they would prove to be *stigmata : i. e.* marks corresponding with the wounds of the nails in the hands of our Saviour ! Shortly afterwards, two other *stigmata* appeared upon her feet, and, about the same time, one also over the region of the heart. Drops of clear blood frequently flowed from these spots on certain days !

Lord Shrewsbury saw her in one of her trances ; she was kneeling, and as motionless as a statue or waxen image. She would have remained in this state for hours ; but her confessor partially *detranced* her by a slight touch or word ; and straightway she fell back upon her pillow, so as to assume a sitting posture. She was, however, still insensible ; and it required her holy father again to address her before she was completely restored to her senses. On awaking, she looked all sweetness, we are told, upon her priest, and kissed his hands with great emotion. As ill-luck would have it, one of the visitors enquired in her hearing, whether the *stigmata* were visible. No sooner were the words uttered, than she at once "became again transfixed in ecstasy."

Was there ever a more complete case of Hysterical catalepsy in a weak exciteable girl than this instance of what Dr. Binns terms "seraphic ecstasy"? It will be observed that a good many of its features are thoroughly Mesmeric.

Let us now hear something of the "Addolorata of Capriana," another of the seraphic ladies. This girl seems to have been in wretched bad health, and subject to attacks of violent pain. As a matter of course, no exact particulars are given. All that we are told is that, during a paroxysm of severe suffering, "one night her head became encircled by small wounds, 53 in number, which opened and bled profusely every Friday. Fourteen days after the Crown of Thorns, she received the *stigmata* in the hands and feet." This poor creature seems to have been subject to convulsive attacks.

We have little doubt that she, like most hysterical girls, morbidly greedy of sympathy, and fond of attracting notice, was in the habit of pricking her forehead and hands with most religious regularity on the appointed days. Be this as it may, our readers will be amused to read his Lordship's report of her appearance.

> "Her hands were firmly clasped over her chest, as one in a state of considerable pain; and her whole frame was convulsed with a short, quick, tremulous motion. The blood was still oozing perceptibly from the wounds in the back of her hands, though the blood and serum, which had flowed from them, did not extend above two, or at most three, inches. Her fingers were so firmly clasped, that to judge from appearances, she had not the power to loose them; but, on the clergyman, who accompanied us, asking her to let us see the inside of her hands, she immediately opened them from underneath, without unclasping her fingers, as a shell opens upon its hinges; so that we distinctly saw the wounds and the blood and serum quite fresh, and flowing down over the wrist. At our request, he also asked the mother to uncover her feet, which she did, though with some small reluctance: when we found them in the same condition as the hands, with, however, this singular and surprising difference, that instead of taking its natural course, the blood flows upwards over the toes, as it would do were she suspended on the cross. We had already heard of this extraordinary deviation from the laws of nature, and were now happy to have an opportunity of verifying it in person."—*Binns*, p. 232.

Lord S. has quite a sharp scent for the discovery of *estaticas*. He found out another in 1842, near Arezzo in Tuscany. We especially request the medical reader's attention to this case, as it seems to us to be a very good specimen of a not uncommon state in young females.

The favoured individual was another girl, as a matter of course, in wretched health, bloodless, and excessively emaciated—in short, as his Lordship says, "a perfect skeleton." When sixteen years old, she was severely hurt by a fall down stairs, and, from the time of this accident, she was liable to attacks of convulsions. For some years past (before the date of the report), she had been entirely bed-ridden. When Lord S. visited her, she was lying calmly on her back, her head being slightly raised, so as to enable her to look, through a grating in the wall, into a small chapel fitted up for her especial service. "At the offertory, she suddenly threw forward the bedclothes, and instantly rose upon her knees with her arms outstretched, and her eyes elevated to Heaven, motionless as a statue. She, who had been bed-ridden for eleven years, and so perfectly helpless that it is with extreme difficulty, as I have just observed, she is moved with the assistance of several persons, and which invariably produces syncope, now sprang into the beautiful attitude in which we then beheld her, with a swiftness, elasticity, and energy, as if a spring of highly-tempered steel were in every joint. She remained in this position for two or three minutes, and, then slowly folding her arms across her breast, she gradually sank again upon her pillow with surprising grace and dignity, and in a manner as unearthly as she had risen. Her mother then covered her again with the counterpane, and she again became conscious of what was passing around her."—*Binns*, p. 241.

Now for the *aerial* phenomena (as they are called by Dr. Binns) witnessed in this poor hysterical girl.

"The chaplain," continues Lord S., "desired me to touch her hand, when the slightest pressure of my finger upon hers made her arm fall several inches, and put her into a swinging motion from side to side. This movement was considerably increased by the same person blowing at her gently with his breath, so exceedingly aerial and unsubstantial is her frame. None of these influences, however, produced the slightest alteration in her attitude, or the least impression upon her senses; her eyes and countenance remaining fixed and immoveable as before, and her arms resting in the same position—only swinging with, and entirely regulated by, the body. Before this motion had entirely subsided, he gently blew at her in front, and she then swung to and fro, backwards and forwards, showing how singularly distinct was the effect of so gentle a breath upon her, the impulse being given according to the direction from whence it came. Gradually the oscillation ceased, and she recovered her balance; when she fell back with the same grace and dignity as upon other occasions, regaining her position upon her pillow with her arms crossed, and was again covered with the counterpane."—*Binns*, p. 242.

So much for the credulity, in the 19th century, not only of a weak-minded nobleman who is a devoted believer in all the legendary fictions of the Popish religion, but of a learned (?) M.D., who—we may here take occasion to remark—has managed to cram into the space of a single volume a greater amount of absurdity than it has ever been our lot to have encountered before. We need scarcely say that, as long as human nature remains the same, fond of the marvellous and supernatural, and as long as hysterical girls are petted and made much of, so long shall we every now and then have accounts of Miraculous cures, Trances, Dreams, Visions, and so forth.

As a matter of course, much depends on outward circumstances what turn the seizure will take, and how the poor invalid will be affected. While, in priest-ridden Italy, two or three sickly anæmic young women are making people (and perhaps themselves too) believe that they are the chosen instruments of Heaven to shew forth—it is not easy to say what; we find at the very same time that there is, in metaphysical Germany, a *Seeress* of *Prevorst*, whose "Revelations concerning the inner life of man, and the inter-diffusion of a world of spirits in the one we inhabit," have just been published by Dr. Kerner of Weinsberg. We need scarcely say that these Revelations are full of the wildest and most extravagant nonsense about demons and all sorts of diablerie.

But we have neither space nor inclination to pursue this subject: our only motive for introducing it at all was that of shewing some of the corporeal effects of inordinately excited feelings in the female system. And here we must not omit to remark, that by far the greater number of cases of Trance, Ecstasy, Somnambulism, &c.—whether spontaneous or the result of Mesmeric Manipulations—have occurred in young unmarried females, subject, in almost every instance, to some one or other of the numerous forms of Hysterical disease. We are ready to admit that there are certain points in the history of these psychico-corporeal conditions, that are not well understood or easily accounted for. Many of the phenomena of Sleep-walking are indeed very strange, nor must we refuse to credit them, because they baffle our attempts to explain their physiology; but certainly we have never yet met with a single indisputable case that would warrant our belief in the alleged existence of Clair-voyance, transposition of the senses, and other unnatural marvels of the Mesmeric Art. Its very advocates have themselves much to blame, by the extravagance and utter senselessness of many of their statements, for not a little of that ridicule and condemnation that have been cast upon it. Witness, for example, the precious production of Mr. Newnham, which we noticed in the last number but one of this Journal.* If medical men commit themselves to such absurdities, we cannot wonder at simple-minded clergymen and barristers floundering about, in the most amusing manner, in a sea, of the ordinary tides and currents of which they are utterly ignorant. No man can be expected to write very rationally on the irregularities of the function of any part, unless he is previously acquainted with its physiology in health, and the changes to which it is liable under the influence of disease; and therefore it is that we can attach but little importance to all the learned labours of the *laity* on the strange and perplexing phenomena of morbid innervation, more especially as they display themselves in hysterical girls. The shrewdest physicians have been puzzled to explain them, and have therefore been content to relate very modestly the facts which they have observed, without attempting to solve or explain them. Cabanis was a man of this description, and it is to him that we are indebted for the following remarks.

"I think it necessary, here, particularly to call to mind those singular acute maladies in which intellectual faculties are suddenly seen to arise and develop themselves, which till that time had not existed. It has been remarked also, in some ecstatic and convulsive affections, that the organs of sense become sensible to impressions which were not perceived in their ordinary state, and even to receive impressions foreign to the nature of man. I have often observed, among

---

* We may take this opportunity of alluding to a very judicious and well-written pamphlet by Mr. Vines of Reading, entitled "Objections to Animal Magnetism or Mesmerism, &c. It clearly shews how Mr. Spencer Hall—one of the itinerant professors of the art—utterly failed in his attempts to hoodwink the good people of Berkshire. Touching these perambulating exhibitors, we recommend some very sensible remarks by Mr. Colquhoun to their notice, at page 177 of his new work on Somnambulism.

women who might have been excellent sorceresses, the most singular effects of the changes of which I speak. There are some of these invalids who easily distinguish microscopic objects with the naked eye; others who see sufficiently clearly in the most profound darkness, to guide themselves with confidence. There are those who follow persons by their track, like a dog, and recognise, by the scent, the objects which these persons have used, or which they have only touched. I have seen those in whom the taste had acquired a peculiar delicacy, and who desired or knew how to choose the food, and even the remedies which appeared really useful to them, with a sagacity which is generally observed only among animals. We see others, who are in a state to perceive in themselves, during the time of their paroxysms, either certain crises which are preparing, and of which the termination proves soon after the justness of their sensations, or other organic modifications attested by those of the pulse and by other signs still more certain."

As an illustration of these remarks we shall here adduce the report of a case related by Dr. Darwin, in his Zoonomia. It occurred in " a young lady, who, after being exhausted by violent convulsions, was suddenly affected by what he calls reverie. She conversed aloud with imaginary persons, her eyes were open, but so intently was her mind occupied that she could not be brought to attend to external objects by the most violent stimulants. The conversations were quite consistent. Sometimes she was angry, at other times very witty, but most frequently inclined to melancholy. Indeed it appears that this reverie only exalted her natural versatility of temper and intellect. She sang with accuracy, and repeated many pages from the poets. In repeating some lines from Pope, she forgot a word, and after repeated trials regained it. In subsequent attacks she could walk about the room, and, although she could not see, she never ran against the furniture, but always avoided obstacles. Dr. Darwin convinced himself that in this state she was *not capable of seeing or hearing* in the ordinary manner. It is observable in this case that volition was not suspended ; she regained by effort the lost word in repeating the poetry, and deliberated according to the natural habit of her mind ; yet, when the paroxysm was over, she could not recollect a single idea of what had passed in it."—*Moore*, p. 103.

The subject of Animal Magnetism naturally enough suggests to the mind the question of Empiricism in general.

What is the cause, we would ask, of so much successful, not to say merely profitable, quackery in the present day? Is it not the vast increase of *malaise*, if not of positive maladies, induced by the highly artificial and unnatural mode of life pursued, and by the consequences of this —inactivity, and self-indulgence? The ready remedy for the pains and aches, and loss of appetite, and indigestion, and vapours, and want of sleep, and so-forth, is to give the patient something to do; and whether this "something" consists in swallowing pills by the dozen at a time, or in taking dainty little globules of sugar at stated hours of the day, avoiding most carefully *this* article of food, and taking most regularly *that* article of drink ; or in being pawed at and stroked down by a nice-looking gentleman ; staring you most fixedly in the face ; or in being galvanised or magnetised, or rubbed, or pummeled, or shampooed ; or in being sodden in steam, or soused in cold water—it matters very little, upon the whole ; any one of them will do equally well, in a vast number of cases, provided the fashion and humour of the day will it to be so. Not but that, in many instances, positive and most serious mischief has been done by the use of some of these pastimes—those, we mean, that are really of an active character; as, for example, the Morrisonian and Priesnitzian methods of cure. A good many individuals have been purged to death by the almost incredible quackeries of the Aberdeen linendraper; and we need scarcely say that, in not a few cases of gouty complaints and visceral diseases, the patient has sacrificed his life to the insane fooleries of the German impostor.

But less violent means are, on the whole, much more successful. All ages have had their charms, and amulets, and incantations to cure, as well as to prevent, diseases. And here it may be worthy of notice that many of these consisted, either in whole or in part, of Music and the recitation of Poetry. Hence, indeed, the very origin of the words *charm* (carmen) and *incantation*. Democritus tells us that many diseases may be cured by the sound of a flute, and Asclepeiades employed the trumpet for the relief of *sciatica*, the pain of which, he says, will vanish whenever the fibres of the affected part begin to palpitate! The custom of wearing Amulets has prevailed in almost every age and country; and doubtless the confidence inspired by the practice has not been without its advantage. And when too was the time that Quacks did not exist? "In the opinion of the ignorant multitude," says Lord Bacon, "witches and impostors have always held a competition with physicians." Galen complains in his day that his patients were more obedient to the injunctions of diviners than to his prescriptions; and which of his successors will not say the same? Let us not be unreasonable; Esculapius and Circe were both children of Apollo. Whatever is mysterious will command the faith of the multitude more than what is known. Pliny knew this well when he said; "minus credunt quæ ad salutem pertinent, si intelligunt."

It is certainly a curious fact in the history of Credulity that rational men and women seem to be so often more ready to surrender their faith and judgment to the most unblushing mountebanks than to their regular medical attendants, who dare not exact one-half of the penances or penalties which the former may impose at their pleasure. But such has been the case at all times and in all countries; and indeed not in medical matters only, but in those of a still higher importance. Does not the devotee of superstition or of the most idolatrous religion willingly submit, at the bidding of his priest, to the most severe inflictions of bodily suffering, to fastings and scourgings, and even to mutilations of the most terrible kind; while the followers of the pure and simple faith are too generally most unwilling to practise abstemiousness even for a single week, or put the slightest check upon their accustomed indulgences? It is one of the besetting sins of man that, when he is ill at ease either in his mind or in his body, he always seeks to be *doing* a great deal himself for the recovery of his wished-for comfort. He is not satisfied with merely following a calm and continued course of passive discipline, mental as well as bodily—the discipline of temperance, regular exercise, and cheerful contentedness:—he wants the excitement of having something to *do*. He is impatient and cannot wait for slow results and the operation of simple means. He would rather be worse than remain in this stationary condition, making no progress to amendment, and not having that degree of sympathy from friends and attendants which a more serious illness would certainly obtain for him. Now what, pray, is the real state of such a patient ?—in point of fact, is it not the mind, rather than the body, that is ill at ease ? If *that* were more occupied and engaged with worthy pursuits, and if there was a larger share of cheerful contentedness and religious submission, *this* would inevitably become less sensitive to every passing ache and uneasiness. As long as there is a vacant and selfish mind, so long will there be an ailing and delicate body.

There is an interesting chapter in the history of Diseases, which of late years more especially, when Solidism has unduly prevailed in nosological enquiries, has been far too much overlooked—we allude to the restorative and healing influence of unassisted Nature. Many maladies, we need scarcely say, have a tendency to cease of themselves, after they have continued for a certain time ; and, unless some of their symptoms exhibit an unusual severity, or an accidental complication happen to exist, the less that Art interferes with the operations of this *vis medicatrix*, the better. Not to mention the family of the Neuroses, to which our attention has been hitherto almost exclusively directed, we might point to the numerous and important class of the Pyrexiæ for many illustrations. In the treatment of them, it is often quite as useful to know when to do very little, as it is when recourse ought to be had to active medication. The days of out-and-out Broussaism are, thank God, passed ; and medical men, even in France, have now found out that a fever is not necessarily an inflammation. The possession of Algeria, if it has not been very useful to our neighbours in a commercial or political point of view, has, at least, had the effect of teaching the medical officers of their army,—and the important lesson has gradually extended itself to the civil practitioners—to abandon many of the principles of their early professional education in Paris, and to have recourse to a more enlightened and successful method of treating the fevers of Africa, which are almost invariably of a remittent or intermittent character. Bark, opium, and wine have, in a great measure, taken the place of venæsection and ptisans. In these fevers, it is of the highest consequence to keep up the spirits of the patient; for the state of the Mind has no inconsiderable influence in aiding or in counteracting the effects of the remedies employed for their subjugation. Often has the expected fit of an Ague been observed not to occur, if the attention has been intensely occupied by something of absorbing interest, or if the feelings have been strongly roused by some joyous or alarming intelligence. A mere effort of the mind, a bold resolution, has been known to prevent the expected paroxysm of an intermittent. This, and some other maladies—especially those of a neuralgic nature—not unfrequently seem to be kept up by the mere force of habit or of long-continued repetition : the suffering is expected at a certain time ; and as sure as it is looked for, so certainly does it return. But we have already alluded to this subject and need not enlarge.

There are still many other topics which might be aptly introduced as illustrating the present subject, were it not for the length to which this article has already extended : we shall therefore mention only one or two that first occur to our notice. Might not the many cures that are effected every year by patients visiting the waters of Bath and Cheltenham, or the Spas of Germany and other places, be adduced, as evidencing the sanative influence of Mind upon Body? We do not mean to deny that the purer air, the more regular exercise, the more temperate meals, and even the imbibition of the medicated waters, have a good deal to do with the invigoration of the health of the invalid; but has not the amusement of the mind, the withdrawal from the carking cares of business or the petty anxieties of fashionable dissipation, and the consequent subsidence of the feelings to a more even and regular flow, *their* share also in the recovery of the jaded appetite, and the relief of the feverish and vexing headache? It is Miss Mitford, we believe, who remarks, in one of her pleasing tales, that whenever we meet with any thing to annoy us, the best plan is to put on our hat or bonnet, and walk out into the open air. Admirable remedy!—its good influence we often feel in our own experience. Whether it be in town or country, we are sure to meet with something out of doors, to engage our attention, and withdraw us from ourselves, and our own vexations and troubles. If we chance to see some sad victim of poverty or disease, in our walk along the street, are we not involuntarily led to compare his condition with our own, and thus to gain a lesson of more submissive equanimity? And where is the man who can look upon the ever-fresh beauties of Nature in the fields, and hear its sweet sounds, and muse on the wonders of Creative skill and goodness around him, without finding some mitigation of his aches and troubles, if it be but for an hour?

And then, how many of the maladies of civilised life arise from sheer inaction, and the utter want of proper exercise either of mind or body! In a large metropolis like this, a vast number of ladies sometimes will not leave the house for a couple of weeks at a time, although their health may be perfectly good all the while. They may not indeed suffer the evil effects of this inaction at the moment; but ere long, if a change be not made, they infallibly become the victims of loss of appetite, constipated bowels, irregular menstruation, leucorrhœa, and such like maladies. And such is the reciprocating and mutually-re-acting influence of body and mind, that an almost inevitable accompaniment of such disordered health is some unamiable change of the Temper. It would be easy to prove this by a variety of examples, and to show how deeply the character, moral as well as mental, is apt to be modified by various maladies that flesh is heir to. The whole work of Cabanis is devoted to the illustration of this very subject, and full of interesting matter it is. We must pass it by at present; for our business is rather to exhibit the influence of Mind upon Body, than of Body upon Mind. But then it may be said; has not the *temper* much to do with the mitigation or aggravation of many maladies? Yes, most assuredly; no one can dispute the fact; for it meets us at every step, and we observe it in patients of all ages. The irritable and passionate child suffers tenfold more in Laryngismus Stridulus and in Pertussis than its fellow of a milder disposition. The importance (in a mere physical point of view) of not indulging children's appetites and inclinations, or giving way to their wayward likings and dislikings, cannot be too much insisted upon. Whoever has seen much of practice in children's diseases, must know well the marked difference as to their obstinacy and duration in different individuals :—arising, too, chiefly from the unmanageableness of some compared with others. Too often we have a double disease to contend with—the malady itself; and the feverish irritability aggravated every time that any medicine is administered.* This remark applies in a more especial manner to the case of cerebral and laryngeal disorders; and not in childhood only, but in riper years, when it might be reasonably expected that the judgment would exercise more controul over the fretfulness of the temper. How often have we seen patients labouring, for example, under an attack of Asthma—in place of being quiet and resigned, striving to moderate the dyspnœa by regularly-repeated acts of as deep inspiration as possible—restless, tossing about from one position to another, now drawing in a deep breath and next moment expelling all the air in the lungs by loud groans and murmurings. Any one, who has been squeezed up in a crowd, knows full well that, if he is at all impatient or quarrelsome, he becomes short-winded, and begins to feel all the horror of impending suffocation. The only plan is to keep quiet, and breathe as evenly as he can. So it is with the poor Asthmatic, during an access of his malady. Medicines are generally of little avail in shortening or relieving it; the chief reliance must be on the exercise of patient endurance. What we have said of Asthma is applicable, in a greater or less degree, to all diseases of the respiratory organs: their severity depends very materially upon the *patience* of the sufferer. The progress of even a disease like Phthisis is often retarded, in a very striking manner, by a spirit of sustained equanimity and resignation. We need scarcely say that it is only the blessed influence of Religion that can impart this inward grace of character. Some years ago we witnessed a beautiful example of this, in the person of a working man. Three years before his death, we ascertained the existence of tubercular disease of the lungs. The course of the malady was most slow and progressive; not a murmur nor sign of impatience was ever manifested by the invalid during the whole time: we always found the Bible open before him. The unusually rapid course, on the other hand, of some cases of Consumption is unquestionably owing, in part at least, to the constant feverishness that is kept up by the irritability and disquietude of the patient's mind.

In drawing these remarks, on the Influence of the Mind upon the Body, to a close, we may be permitted to quote a passage from the review of an interesting paper on "Moral Therapeutics," by the accomplished Secretary of the Royal Academy of Medicine in Paris, contained in the Number of this Journal for October, 1841.

"There cannot be a doubt but that psychological causes of disease are too apt to be entirely overlooked in the present day, and that physicians, in their minute examination of all the physical symptoms of a malady, often neglect the influence of mental emotions on its development, its progress, and its termination. 'If a patient dies,' says M. Reveillé-Parise, 'we open his body, rummage among

---

* It is often asserted that the treatment of the diseases of infants and children is much more difficult than that of adults, in consequence of the former being unable to explain their disordered feelings so well as the latter. True; but then there is not the perturbing influence of mind and feelings in the one case to baffle and counteract the operation of our remedies. There may be *temper;* but the effects of that are very different from those of fear, anxiety and so forth.

the viscera, and scrutinize most narrowly all the organs and tissues, in the hope of discovering lesions of some one sort or another; there is not a small vessel, membrane, cavity, or follicle, which is not attentively examined; the colour, the weight, the thickness, the volume, the alteration, nothing escapes the eyes of the studious anatomist. He handles, touches, smells and looks at every thing; then he draws his conclusions one way or the other. One thing only escapes his attention; this is, that he is looking at merely organic effects, forgetting all the while that he must mount higher up to discover their causes. These organic alterations are observed, perhaps, in the body of a person who has suffered deeply from mental distress and anxiety; these have been the energetic cause of his decay; but they cannot be studied in the laboratory or in the amphitheatre.' * * * * * 'Many physicians of extensive experience are destitute of the ability of searching out and understanding the moral causes of disease; they cannot read the book of the heart; and yet it is in this book that are inscribed, day by day and hour by hour, all the griefs, and all the miseries, and all the vanities, and all the fears, and all the joys, and all the hopes of man, and in which will be found the most active and incessant principle of that frightful series of organic changes which constitute pathology.'

"This is quite true,—whenever the equilibrium of our moral nature is long or very seriously disturbed, we may rest assured that that of the animal functions will suffer.

"Many a disease is the *contre-coup*, so to speak, of a strong moral emotion; the mischief may not be apparent at the time, but its germ will be nevertheless inevitably laid."

It only remains for us now briefly to notice the general character of the books, whose titles we have prefixed to this article. The work of Dr. Moore contains a good deal of interesting information, and is imbued throughout with an amiable and enlightened spirit of religious feeling. That of Dr. Binns is utterly unworthy of any man of education: doubtless it is intended for the eye, not of medical men, but of the reading public; and probably the Doctor has had his reward. The attempt to impose upon the credulity of people by pretending—at a personal sacrifice too!—to communicate a ready means of procuring sleep at any time, is truly disgraceful on the part of a regularly qualified physician. The work of Dr. Boismont, on the subject of Hallucinations, is almost entirely occupied with the relation of cases illustrative of almost every sort of monomaniacal delusions. It may therefore be regarded rather as a *Catalogue Raisonnée* of reports, than as a treatise upon the subject. It is unnecessary to say any thing of the "Rapports, &c." of Cabanis, originally published at the beginning of the present century, further than to recommend the present edition to the notice of our readers: it is very complete, and at the same time most reasonable in price. Mr. Colquhoun's new publication is a curious one. Besides the seven lectures of Dr. Wienholt, translated from the German, there is a long Preface and Introduction denunciatory of medical men for their ungenerous opposition to the claims of the noble science of Animal Magnetism, and also an Appendix, in which there is ample reference to (what Mr. C. considers) indubitable cases of Clairvoyance, &c. The lectures of Wienholt were published upwards of forty years ago; and it is not very obvious what could have induced the learned advocate to have disinterred them from their quiet repose. They are full, as might be expected, of that German spirit of most unsatisfying disquisition which leaves the reader at the close just about as wise as he was at the commencement; reminding us more than once of the witty Frenchman's remark on a similar subject; "plus il y a de raisonnement, moins il y a de raison." One of the strongest arguments adduced by him is this. Bats can guide themselves when their eyes have been scooped out as well as when they had them, poor creatures, in their heads; and Cats and Owls can see not only as well, but even better, in the dark than in the light. Here then are proofs—that the faculty of Vision in animals is not confined to the eye-balls! Why then should it necessarily be so in the human being?—more especially when we call to mind the numerous examples where Somnambulists have been known to see objects, although their eyes were perfectly closed at the time. This seems to be the drift of the author's reasoning. The only remark that we shall make is, that it certainly seems rather strange that the Creator should have made a special organ for sight, if animals can see as well without as with it.

The learned translator dwells forcibly upon what may be called a spiritual view of the question, and appeals to the writings, not only of metaphysicians but also of St. Paul, in confirmation of his views. The following passage from Dr. Reid is deemed so conclusive by him that it is actually printed in capital letters throughout.

"We have reason to believe, that when we put off these bodies, and all the organs belonging to them, our perceptive powers shall rather be improved, than destroyed or impaired. We have reason to believe that the Supreme Being perceives every thing in a much more perfect manner than we do, without bodily organs. We have reason to believe that there are other created beings endowed with powers of perception more perfect and more extensive than ours, without any such organs as we find necessary."

The quotation, which he adduces from Scripture in support of his doctrine that perception may exist independent of any bodily organs, is, to say the least of it, any thing but happy for his orthodox theology. After citing the well-known verses from the first Epistle to the Corinthians—"there are celestial bodies, and bodies terrestrial;" and again, "it is sown a natural body, it is raised a spiritual body,"—he adds: "Man, in short, is composed of two different natures—the physical or corporeal, and the psychical or spiritual. In our present state, these two natures are conjoined; in the future state, the soul or spirit will be disembodied, and our knowledge, instead of being acquired through the instrumentality of material organs, will probably be derived from immediate intuition—the product of pure consciousness." Is not this conjecture somewhat at variance with the scriptural doctrine of the Resurrection of our *real* bodies?

By the bye, Mr. Colquhoun is very severe against Dr. Elliotson and his friend Dr. Engledue for their materialist and infidel opinions. He seems to regard the former of these gentlemen as altogether very shallow in his mesmeric, as well as in his other attainments. "The learned doctor," he remarks, "is continually carried off his feet by every novelty—his mind (if any mind he has) is eminently disqualified for philosophical research—when he happens to get hold of any truth, he seems incapable of making a right use of it:—he seems incapable of distinguishing truth from illusion. A considerable part of his letter (appended to Dr. Engledue's very absurd Address to the Phrenological Association) is taken up with an account of certain experiments in that new *bastard science* called Phreno-Mesmerism, of which he speaks in terms of the most rapturous delight." This language (and there is more of it in a similar strain) is surely very unbecoming, applied to a man of Dr. Elliotson's intellectual powers. That he has erred, and erred very grievously, in giving currency to materialist views in his physiological writings, we readily admit; but there are many points in his character that deserve praise. He is sincere and ingenuous, and withal courageous and unhesitating in the avowal of his opinions.

Whether Mr. Colquhoun's mind is "eminently qualified for philosophical research," we shall leave the reader to judge for himself, after he has read what may be called the peroration of the appendix to these lectures.

"If we admit the transposition of the poles of sensibility in somnambulism, by which the central point of the sensitive life is transferred from the brain to some other organ, it is not clear why, as in metastatic affections, sometimes one, sometimes another organ, but always that most susceptible of disease, is attacked —so, in the case of somnambulism, a different organ of the nervous system—especially that most susceptible of zoo-magnetic action—may not be elevated to the dignity of an organ of the soul. If we once assume, so to speak, a *metempsychosis* in the sphere of our own body, (without which, do as we will, we cannot attain to any physiological principle in this matter,) then, under certain conditions, any part of the nervous system may become an organ of the soul, viz. always that part which is most exciteable; and the organ of the soul can thus be transferred out of the central part of the brain into a peripheric part of it, as into the great ganglionic *plexus* of the abdomen, or into any other ganglion, provided this part of the nervous system stands in the relation of a centre, although in a lower sphere, in subordination to the brain—that is, provided it be really a ganglion in the physiological sense. Hence, perhaps, a difference might take place between male and female somnambulists; seeing that in the latter, in consequence of the greater activity of the abdominal ganglions, even in the normal state, most nervous diseases are reflected, and somnambulism, also, most frequently developed in that sex."—P. 200.

